# Alignment and Position Sensors Based on Split Ring Resonators

**DOI:** 10.3390/s120911790

**Published:** 2012-08-29

**Authors:** Jordi Naqui, Miguel Durán-Sindreu, Ferran Martín

**Affiliations:** CIMITEC, Departament d'Enginyeria Electrònica, Universitat Autònoma de Barcelona, 08193 Bellaterra (Barcelona), Spain; E-Mails: miguel.duransindreu@uab.cat (M.D.-S.); ferran.martin@uab.cat (F.M.)

**Keywords:** split ring resonators, coplanar waveguide, alignment sensors, position sensors

## Abstract

In this paper compact alignment and position sensors based on coplanar waveguide (CPW) transmission lines loaded with split ring resonators (SRRs) are proposed. The structure consists of a folded CPW loaded with two SRRs tuned at different frequencies to detect both the lack of alignment and the two-dimensional linear displacement magnitude. Two additional resonators (also tuned at different frequencies) are used to detect the displacement direction. The working principle for this type of sensor is explained in detail, and a prototype device to illustrate the potential of the approach has been designed and fabricated.

## 1. Introduction

**Figure 1.**
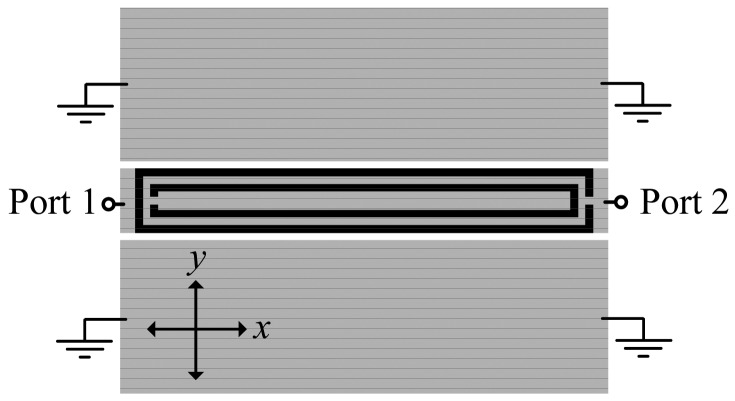
Typical topology of an angular or linear displacement sensor consisting of a CPW transmission line loaded with an SRR (in black) etched in the back side of the substrate. A rectangular shaped SRR is etched just below the central strip of the CPW to improve the sensitivity performance.

This paper is a continuation of a previous paper published by the authors [1], where, for the first time, the symmetry properties of split ring resonators (SRRs) were considered to detect displacement (many other works where SRRs are used for sensing purposes have been reported in the literature [2–8]). In [1], we reported a displacement sensor, able to detect angular or one-dimensional linear displacement. The structure, shown in Figure 1 for completeness, is a coplanar waveguide (CPW) loaded with a single SRR etched in the back side of the substrate. If the SRR is symmetrically etched as in Figure 1, the particle (SRR) cannot be excited and signal transmission between the input and the output port is allowed. However, if the symmetry is broken, for instance by either a rotation or a lateral linear displacement (*i.e.*, along the *y*-axis), the SRR is excited, and a notch arises in the transmission coefficient at SRR resonance which depends on the displacement. The resulting sensitivity (45 dB/mm in average value for linear displacement) and its linearity are reasonable, relative to the considered CPW and SRR dimensions (in the millimeter-scale). The sensitivity was defined as the variation of the notch magnitude of the transmission coefficient with the variable to be sensed, that is, the displacement. However, the structure of Figure 1 is not able to detect a relative displacement between the SRR and the CPW in the axial direction of the CPW (*i.e.*, the *x*-axis). Moreover, it is not possible to distinguish between lateral displacements in the two possible directions (*i.e.*, ±*y*). In this paper, we provide a solution to these two issues. In Section 2, the proposed sensing device is presented, and the working principle is explained in detail. The design strategy, the fabricated prototype device, and the characterization of this prototype are reported in Section 3. Finally the main conclusions of the paper are highlighted in Section 4.

## 2. The Proposed Sensor and Principle of Operation

It is well known that an SRR can be excited by means of an axial time varying magnetic field [9]. Nevertheless, an SRR exhibits cross polarization, that is, it can also be driven by means of an electric field confined in or parallel to the plane of the particle and orthogonal to the plane containing the slits. In [10,11], it was demonstrated that by loading a CPW transmission line with pairs of SRRs, that is, with the centers of the SRRs aligned with the slots of the CPW structure, these particles are excited and a stop band in the transmission coefficient arises. The excitation of the resonators comes from the coupling of the rings with the magnetic and electric field generated by the currents flowing on the CPW. However, if single SRRs (rather than pairs) are symmetrically etched in the back side of the line (as Figure 1 illustrates), the magnetic and electric field components inside the SRR exactly cancel, and the particle is not driven at the fundamental resonance. This situation does not hold if the symmetry is broken, and therefore, a possible lack of alignment or relative lateral displacement between the line and the SRR can be detected and measured, respectively, as was shown in [1]. Provided the symmetry plane of the SRR does not extend beyond the CPW slots, the larger the lateral shift, the deeper the notch in the transmission coefficient. Therefore, the lateral displacement range will be limited to the distance between the two slots of the considered CPW.

In order to extend the sensing capability to two dimensions for alignment and linear displacement, one possible strategy is to introduce a right angle bend in the CPW transmission line, and to etch an SRR in each CPW section. Obviously, these SRRs must be tuned at different frequencies in order to discriminate between displacement in the *x*- or *y*-axis. However, by these means it is not possible to distinguish between up or down and right or left shift in the *y* and *x* orientation, respectively. Our proposal to detect the displacement direction consists of introducing two additional resonators, one in the *x*-oriented CPW section and the other one in the *y*-oriented section, both etched also in the back side of the substrate, but situated beneath one of the CPW ground planes, near a CPW slot. If the displacement direction drives such additional resonators towards the slot of the CPW, this will be detected by a notch at the resonance frequency of these resonators. Conversely, by shifting the SRRs in the opposite direction such notch will not appear. Since it is necessary that the four required SRRs are tuned at different frequencies, the resonators dimensions must be different. Notice that these two additional resonators are introduced to simply detect the displacement direction (they do not provide information on the displacement magnitude). Therefore, we can call these SRRs as direction sensing resonators, to differentiate them from the displacement sensing resonators, those which measure the linear displacement magnitude.

**Figure 2.**
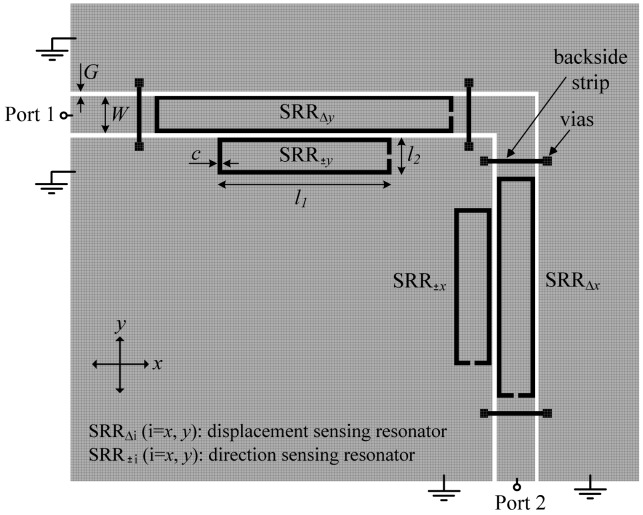
Layout of the proposed sensing device for the aligned position (*i.e.*, the CPW and the displacement sensing SRRs are aligned). The CPW strip and slot widths are *W* = 1.67 mm and *G* = 0.2 mm, respectively, the vias have a 0.2 mm radius, and the narrow strips between vias have a width of 0.2 mm. The dimensions of the SRRs are: *l_1_* (SRR_Δ_*_x_*) = 9.95 mm, *l_1_* (SRR_±_*_x_*) = 7.05 mm, *l_1_* (SRR_Δ_*_y_*) = 13.4 mm, *l_1_* (SRR_±_*_y_*) = 7.8 mm, *l_2_* = 1.67 mm, and *c* = 0.2 mm.

The layout of the designed sensor is depicted in Figure 2. The considered substrate is the *Rogers RO3010* with dielectric constant *ε_r_* = 10.2, thickness *h* = 127 μm, and loss tangent tan*δ* = 0.0023. As discussed in [1], narrow substrates are necessary to boost the sensitivity. For these very narrow substrates, the coupling between the inner and the outer ring of the SRR (Figure 1) is negligible. In the present work single ring SRRs are considered, whose second resonance frequency is located beyond the one of SRRs with two rings [12], and this is important to avoid interference between the transmission notches of different resonators. The vias and backside strips are used to connect the ground plane regions and thus prevent the appearance of the CPW parasitic slot mode.

**Figure 3.**
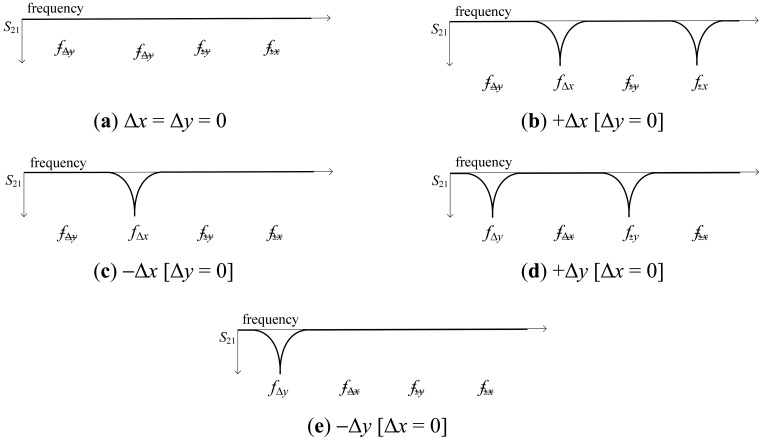
Scheme indicating the primitive shifting operations and the resulting transmission coefficient *S*_21_. A notch is indicative of an SRR excitation. A linear displacement in the *x*- and *y*-orientation is indicated as Δ*x* and Δ*y*, respectively, relative to the aligned position (*i.e.*, Δ*x* = Δ*y* = 0).

**Figure 4.**
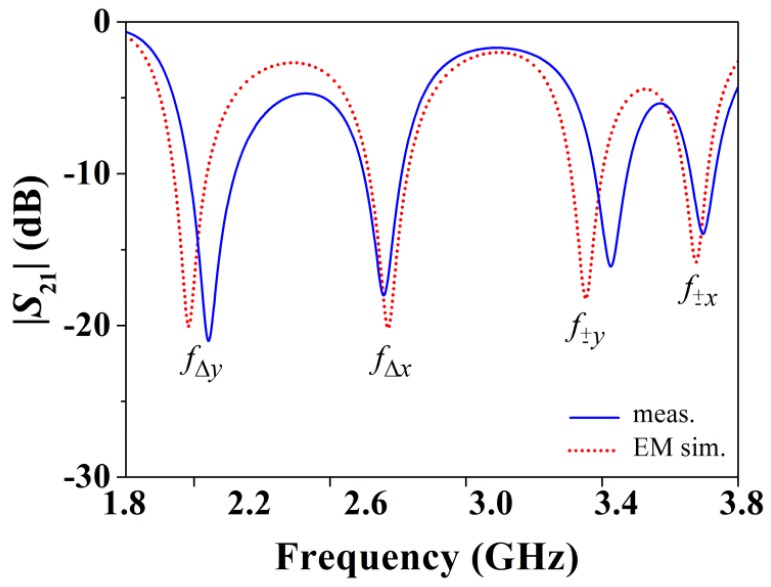
Transmission coefficient *S*_21_ of the sensor for Δ*x* = 0.3 mm and Δ*y* = 0.25 mm.

For a better comprehension of the principle of operation of the proposed sensor, let us consider the four different displacements indicated in Figure 3 from the aligned structure, that is, right, left, up, and down displacements. The resonance frequencies of the four SRRs are denoted as *f*_Δ_*_y_, f*_Δ_*_x_, f*_±_*_y_*, and *f*_±_*_x_* (see Figure 2). It can be seen that displacements in the ±*x*- and ±*y*-direction can be detected (by means of the resonators SRR_±_*_x_* and SRR_±_*_y_*) and measured (by the resonators SRR_Δ_*_x_* and SRR_Δ_*_y_*). Any other linear displacement is a combination of the previous ones, and hence it can also be detected and measured. As an illustration, Figure 4 shows the obtained transmission coefficient for Δ*x* = 0.3 mm and Δ*y* = 0.25 mm.

## 3. Results

**Figure 5.**
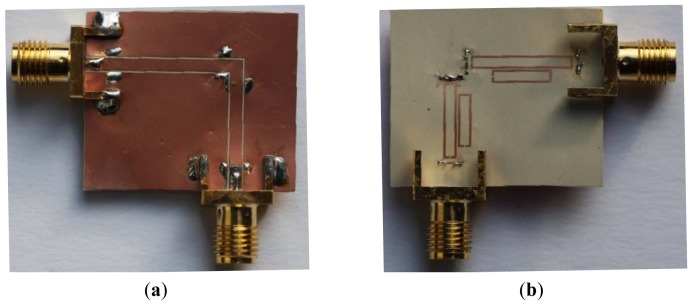
Photograph of the proposed device for the aligned position; (**a**) top and (**b**) bottom face.

The proposed sensing structure is validated by considering several proof-of-concept prototypes with different displacements, by measuring the frequency responses and by representing the notch magnitude of the different involved resonance frequencies (Figure 5 shows a photograph of the device for the case Δ*x* = Δ*y* = 0). Obviously, in a real sensor, the SRRs must be etched on a different substrate in order to achieve relative motion between the sensing SRRs and the bended CPW transmission line, but this complicates the measurement (from the mechanical viewpoint) and, for this reason, by the moment, we have proceeded in this way. In order to validate the proposed approach, we have considered positive and negative displacement in the *x*-direction (horizontal shift), as well as in the *x* = *y*-direction (diagonal shift). This is representative of the potentiality and validity of the proposal.

**Figure 6.**
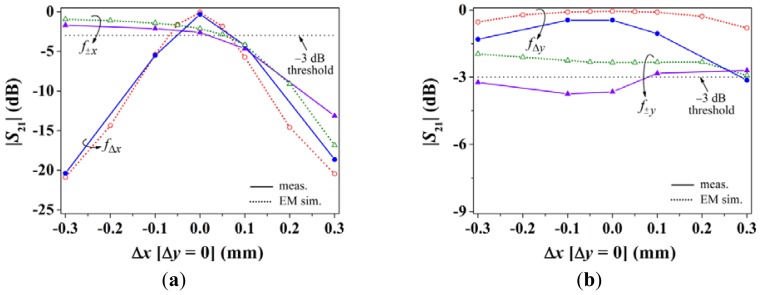
Notch magnitude of the transmission coefficient *S*_21_ at the indicated frequencies for *x*-oriented displacement; results for (**a**) *x*- and (**b**) *y*-axis position sensing.

Figure 6 depicts the dependence of the notch magnitude (simulated and measured) with displacement in the ±*x*-direction. The measurements have been inferred by means of the *Agilent* E8364B vector network analyzer, whereas the simulations have been carried out by means of the *Agilent Momentum* commercial software. As expected, for positive displacements, the SRR_±_*_x_* is activated as is manifested by a clear increase in the notch at *f*_±_*_x_*, whereas the specified −3 dB threshold level is not exceeded for negative displacements (indicating that the shift is in the negative direction). The dependence of the notch magnitude for *f*_Δ_*_x_* is similar and roughly linear in both directions, with a measured value of approximately −20 dB for Δ*x* = ±0.3 mm, which is indicative of a significant sensitivity of roughly 65 dB/mm (average value). On the other hand, the notch corresponding to *f*_Δ_*_y_* is approximately 0 dB, which indicates that the structure is aligned with the *y*-axis (for Δ*x* = 0.3 mm the notch is slightly above −3 dB because for this sample the *y*-axis position sensing resonators are somewhat misaligned due to fabrication tolerances and under-etching).

**Figure 7.**
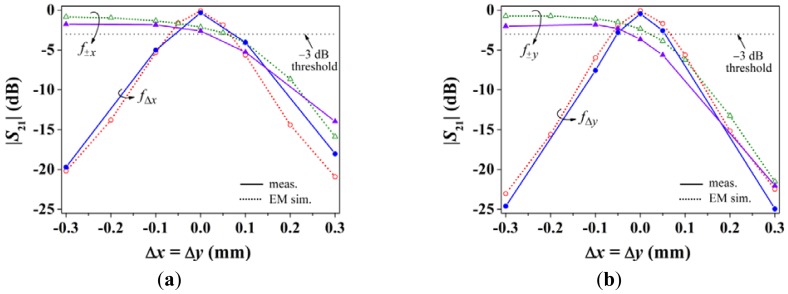
Notch magnitude of the transmission coefficient *S*_21_ at the indicated frequencies for *x* = *y*-oriented displacement; results for (**a**) *x*- and (**b**) *y*-axis position sensing.

The results of the relative displacement in the diagonal orientation (Δ*x* = Δ*y*) are depicted in Figure 7. Similar conclusions to those pointed out for *x*-motion can be inferred to the light of this figure. Nevertheless, it is worth mentioning that the notch magnitude associated to a displacement sensing resonator depends not only on the displacement, but also on inter-notch interference and resonator dimensions. This causes that, for the same displacement, the notch magnitude of the *y*-axis displacement sensing resonator produces a deeper notch than that of the *x*-axis. With these results, the proposed alignment and two-dimensional linear displacement sensing structure is validated.

Another two-dimensional displacement sensor based on split ring-loaded lines was reported in [13]. The operation principle of that approach is founded on the shift of the resonance frequency by using triangular complementary split rings in Microstrip technology. Since external (ambient) conditions may affect the resonator resonance frequency but not the transparency of the lines with perfectly aligned resonators, the proposed sensor of this work is more robust and specially suited for alignment purposes.

## 4. Conclusions

In conclusion, an alignment and a two-dimensional linear displacement sensor device based on the symmetry properties of SRRs has been proposed and validated. The sensing mechanism is based on the electromagnetic coupling between a CPW transmission line and an array of SRRs. The device is able to detect the lack of alignment and the relative linear displacement between two surfaces, one containing the SRRs tuned at different frequencies, the other one including the CPW used to identify the displacement magnitude and direction (by measuring the transmission coefficient). The position sensor characterization has revealed that two-dimensional linear displacement with reasonable sensitivity and linearity can be measured. This represents a significant progress as compared to the former approach reported in [1].
